# Propolis–xenograft scaffolds: comparing delivery methods for tibial bone regeneration in rats

**DOI:** 10.2340/biid.v13.46215

**Published:** 2026-07-02

**Authors:** Yunia Dwi Rakhmatia, Lisda Damayanti, Rasmi Rikmasari, Nuroh Najmi, Sri Tjahajawati, Eriska Riyanti

**Affiliations:** a Department of Prosthodontics, Faculty of Dentistry, Universitas Padjadjaran, Bandung, Indonesia; b Department of Oral Biology, Faculty of Dentistry, Universitas Padjadjaran, Bandung, Indonesia; c Department of Pediatrics Dentistry, Faculty of Dentistry, Universitas Padjadjaran, Bandung, Indonesia

**Keywords:** Propolis extract, xenograft, bone regeneration

## Abstract

**Background:**

Alveolar bone loss after tooth extraction may compromise implant placement and often requires regenerative treatment. Propolis, a natural compound with osteogenic and anti-inflammatory properties, may enhance bone regeneration, particularly when combined with xenograft biomaterials. This study evaluated the effects of oral and topical propolis, alone and in combination with xenograft, on bone healing in a rat tibial defect model.

**Materials and methods:**

Sixty male Wistar rats were randomly assigned to six groups (*n* = 10): control, oral propolis, topical propolis, xenograft, oral propolis with xenograft, and topical propolis with xenograft. Standardized tibial defects (5 × 2 × 2 mm) were created and treated accordingly. After 4 weeks, radiographic, histological, and histomorphometric analyses were performed. Outcomes included relative bone density, new bone area, and osteoblast and osteoclast counts. Data were analyzed using one-way ANOVA with Tukey post hoc test (*p* < 0.05).

**Results:**

Relative bone density increased from 19% in the control group to 81% in the topical propolis with xenograft group (*p* < 0.05). This group also showed the largest new bone area (434,196 ± 153,911 µm² vs. 89,408 ± 42,376 µm²; *p* < 0.001), higher osteoblast counts (178.4 ± 44.2 vs. 49.4 ± 15.2), and lower osteoclast counts compared with single-treatment groups (*p* < 0.001). Combination treatments, particularly topical propolis with xenograft, consistently yielded superior outcomes (*p* < 0.01).

**Conclusion:**

Propolis enhances bone regeneration, especially when applied topically in combination with xenograft. This approach promotes bone formation and balanced remodeling, supporting its potential for clinical application in bone regenerative therapy.

## GRAPHICAL ABSTRACT

**Figure:**
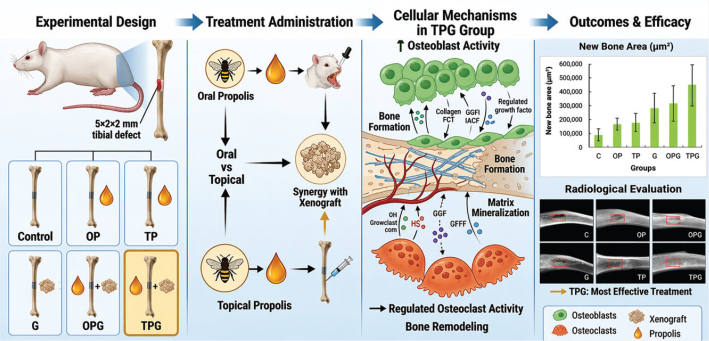

## Introduction

Tooth loss is a significant global public health concern, affecting function, appearance, and mental well-being [1]. Loss of teeth leads to biological changes in surrounding tissues, particularly the alveolar bone. Following extraction or injury, the alveolar ridge undergoes horizontal and vertical bone loss due to the absence of mechanical stimulation from the periodontal ligament [2, 3]. Most bone loss occurs within 6 months after extraction, complicating future prosthetic or implant treatments [4, 5].

Dental implants are the preferred solution for replacing missing teeth, offering durability and restoration of function and appearance. Long-term studies report success rates exceeding 95% under optimal conditions [5, 6]. However, adequate bone volume and quality are essential for implant stability and integration [6]. Bone defects from trauma, periodontal disease, or prolonged tooth loss often necessitate regenerative procedures before implant placement [2].

Bone regeneration is a complex process involving bone-forming cells, signaling molecules, and the surrounding matrix [7]. It occurs in overlapping stages: inflammation, angiogenesis, bone formation, and remodeling [8]. Osteoblasts build bone by producing collagen and initiating mineralization, while osteoclasts resorb bone during remodeling [7]. Effective bone healing requires a balance between these cell types.

Treatment of bone defects includes guided bone regeneration, growth factor therapy, and bone grafting. Bone grafts are widely used in dentistry to support new bone growth and provide structural support [9]. Grafts may be autografts, allografts, xenografts, or alloplastic materials [9]. Bovine bone xenografts are commonly used due to their availability and their similarity to human bone, which facilitates new bone cell growth [10]. These xenografts serve as scaffolds, supporting cell migration and maintaining bone shape.

While xenografts are widely used, they mainly act as passive scaffolds and may not actively promote new bone growth [11]. Recent research has focused on combining bone grafts with biological agents to enhance cell activity and accelerate regeneration [12]. Natural products are gaining interest in regenerative medicine due to their active compounds and lower toxicity [13].

Propolis is a sticky substance that honeybees collect from plants and mix with wax and enzymes. Its composition varies by source but typically includes flavonoids, phenolic acids, terpenoids, and aromatic compounds [14]. Studies indicate that propolis has antimicrobial, antioxidant, anti-inflammatory, and immune-modulating properties [14]. These features make it a promising candidate for medical applications, including wound healing and bone regeneration [14]. Studies suggest that propolis can affect bone metabolism by promoting osteoblast growth and development while slowing osteoclast activity [15]. The flavonoids in propolis are known to trigger pathways that help form bone and make collagen [16]. Its antioxidant effects may also reduce oxidative stress during healing, thereby supporting tissue repair [17]. These features suggest that propolis could enhance bone healing when combined with bone grafts.

Propolis can be administered systemically intra-oral or locally at the defect site [17, 18]. Systemic delivery distributes bioactive compounds throughout the body, but metabolism may reduce their concentration at the target tissue. Topical application delivers propolis directly to the defect, potentially achieving higher local concentrations and stronger biological effects [18, 19].

Previous studies show that propolis enhances bone regeneration by stimulating osteoblast activity, increasing bone density, and accelerating healing of bone defects and fractures [19, 20]. However, limited data exist on the optimal method of propolis administration with xenograft materials. This study compared the effects of oral and topical propolis extracts, combined with xenograft, on bone regeneration in rat tibial defects. Bone healing was evaluated using radiographic and histological methods, including bone density measurement, osteoblast and osteoclast quantification, and analysis of new bone area. Determining the most effective administration route is essential for optimizing regenerative strategies in implant dentistry. This study, therefore, aimed to evaluate and compare the effects of oral and topical propolis, alone or in combination with xenograft, on bone regeneration in a standardized experimental model.

## Materials and methods

### Experimental animals

Sixty male Wistar rats (*Rattus norvegicus*), aged 6 weeks and weighing 200–250 g, were obtained from a certified facility. Animals were acclimatized for 7 days under standardized conditions (22–25°C, 50–60% relative humidity, 12-h light/dark cycle) with free access to standard diet and water. Only healthy rats were included; those with >10% weight loss or signs of illness during acclimatization were excluded. All procedures were approved by the Institutional Animal Ethics Committee of Universitas Padjadjaran (No. 55/UN6.KEP/EC/2025) and adhered to the 3Rs (Replacement, Reduction, and Refinement) and the Five Freedoms of animal welfare.

Animals were divided into six groups (*N* = 10 rats per group): control defect without treatment (C), oral propolis (OP), oral propolis combined with xenograft (OPG), topical propolis (TP), topical propolis combined with xenograft (TPG), and xenograft-only group (G). Propolis extract (EFI Propolis, Kabanjahe, Karo Regency, North Sumatra, Indonesia) was obtained from *Geniotrigona thoracica* stingless bees and prepared using 70% ethanol extraction, yielding a total phenolic content of 124.6 ± 1.5 mg GAE/g and total flavonoid content of 42.0 ± 0.8 mg QE/g dry weight [21]. A daily dose of 0.025 mL was administered to the OP and OPG groups, calculated according to the Laurence and Bacharach method, with its bioactive flavonoid and phenolic compounds serving as the basis for its therapeutic application in this study [22]. Oral administration was performed daily via pipette directly into the oral cavity for 4 weeks, while topical application was applied once directly to the defect site during surgery in the TP and TPG groups. Xenograft material (Bovine-derived bone powder; Batan Graft, PT Focustindo Cemerlang, Bogor, Indonesia) was applied at a standardized dose of 2 mg per defect. All animals were assigned to treatment groups using simple randomization with a random number table.

### Surgical procedure

All surgical procedures were conducted under aseptic conditions. General anesthesia was administered using a standardized protocol, followed by a longitudinal incision along the medial tibia. Soft tissues were retracted to expose the bone. Blood samples were collected at this stage to measure blood glucose and hemoglobin levels. Glucose was measured with a calibrated glucometer, and hemoglobin was measured with a commercial assay kit per the manufacturer’s instructions. Measurements were taken before defect creation (baseline) and after the 4-week healing period, immediately before euthanasia. Mean values were calculated for analysis. These assessments controlled for systemic factors that could influence bone healing, such as hyperglycemia and altered oxygen-carrying capacity, which can affect osteogenesis, angiogenesis, and tissue regeneration.

A standardized defect (5 × 2 × 2 mm) [23] was created in the mid-diaphysis of the right tibia using a low-speed rotary instrument with diamond burs. Continuous saline irrigation was used to prevent thermal injury. Soft tissue trauma was minimized, and defect size was kept consistent across all animals. Treatments were then applied according to group allocation. Propolis extract was administered at 0.025 mL per day orally for the oral groups (OP and OPG), while the topical groups (TP and TPG) received a single topical application of 0.025 mL directly to the defect site during surgery. All surgical sites were sutured using sterile technique following treatment application.

### Radiographic analysis

After 4 weeks, animals were euthanized with an overdose of anesthetic agents, and tissues were harvested. Tibial specimens underwent radiographic and histological evaluation. Radiographs were obtained with a portable X-ray system and analyzed in ImageJ to quantify relative bone density (RBD) [24]. The region of interest (ROI) at the defect site was compared to adjacent normal bone using mean gray values to provide a standardized measure of bone regeneration.


$$\text{Relative Bone Density }\left(\%\right)=\frac{\text{Mean gray value }\left(\text{defect}\right)}{\text{Mean gray value }\left(\text{normal bone}\right)}\times 100$$

### Histological and histomorphometric evaluation

Specimens were fixed in formalin, dehydrated in graded alcohol, cleared in xylene, and embedded in paraffin for histological evaluation. Serial sections were stained with hematoxylin and eosin (H&E). Histomorphometric analysis was performed using a light microscope at 40× magnification to measure new bone formation area. Three random, non-overlapping fields per section were analyzed at 100× magnification to quantify osteoblasts and osteoclasts. The mean of these three fields was used for statistical analysis. Histological and histomorphometric evaluations were conducted by an evaluator blinded to group assignments.

### Statistical analysis

Primary outcome measures included RBD, new bone formation area, and the number of osteoblasts and osteoclasts as indicators of bone remodeling activity. All data were expressed as mean ± standard deviation. Statistical analysis was performed using one-way ANOVA followed by post hoc Tukey test, with a significance level set at *p* < 0.05.

## Results

### Relative bone density

**Figure 1:**
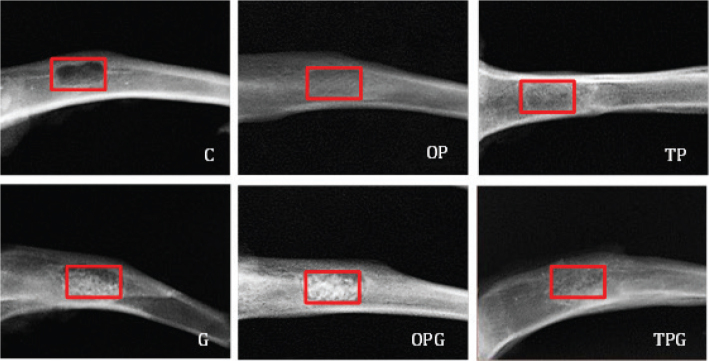
Radiographic comparison of bone density among experimental groups (red square shows ROI).

Radiographic assessment of the region of interest was conducted after a 4-week healing period following tibial bone defect creation in rats (Figure 1). The C group showed minimal healing, with low bone density, a visible defect, and limited callus formation. The OP and TP groups exhibited increased bone density and partial defect filling, as seen by more homogeneous radiopaque areas and early bone bridging. The G, OPG, and TPG groups demonstrated improved bone regeneration, with 60–70% reductions in defect size, thicker callus formation, and partial restoration of tibial cortical continuity compared with the control.

**Figure 2:**
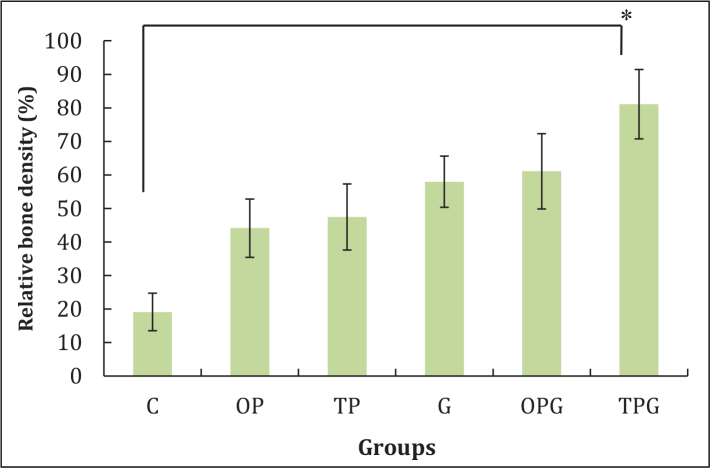
Relative bone density of experimental groups. A significant difference between C and TPG group (*p* < 0.05).

Radiographic analysis showed a progressive increase in RBD in all treatment groups compared to the control (Figure 2). Mean RBD values (%) for the C, OP, TP, G, OPG, and TPG groups were 19.11, 44.13, 47.47, 57.97, 61.11, and 81.09, respectively. The C group had the lowest bone density, indicating minimal regeneration, while the TPG group had the highest, indicating enhanced osteogenesis. The OP, TP, G, and OPG groups showed intermediate improvements. Statistical analysis confirmed a significant difference between the C and TPG groups (*p* < 0.05), demonstrating the superior effect of the combined treatment.

### Histological findings

**Figure 3:**
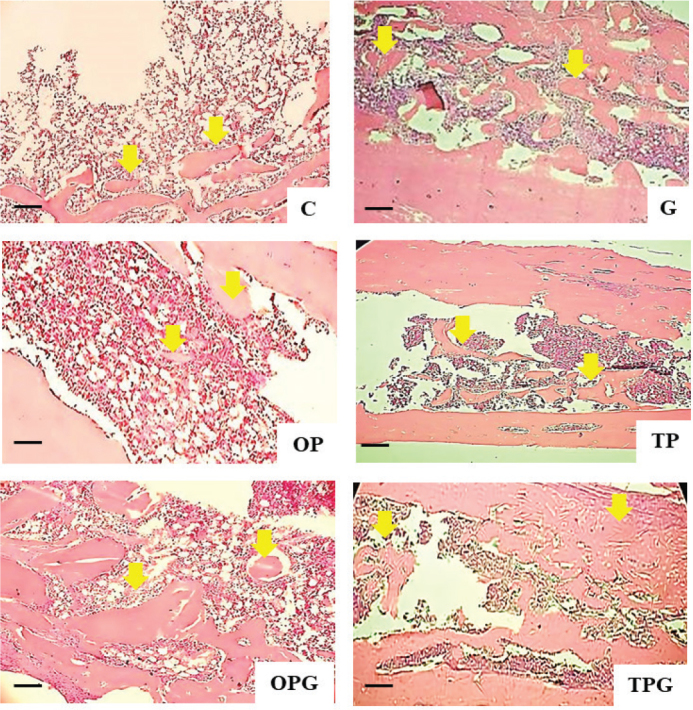
Histological sample appearances of experimental groups after a 4-week healing period at 40× magnification. Yellow arrows indicate areas of new bone. (Scale bar: 100 µm).

Histological analysis at 4 weeks post-treatment demonstrated marked differences in bone regeneration among the experimental groups (Figure 3). The C group showed minimal new bone formation, characterized by sparse trabeculae and predominantly fibrous connective tissue within the defect area. In the OP group, an increase in newly formed bone was observed, with more evident trabecular structures and early osteoid deposition compared to the control, indicating enhanced osteogenic activity. The TP group exhibited more pronounced bone formation than the OP group, with thicker and more organized trabeculae, suggesting that local application may provide a more direct stimulatory effect on the defect site. In the G group, histological evaluation revealed moderate new bone formation with partial defect filling and visible integration of graft particles. The most substantial bone regeneration was observed in the combination groups, particularly in the TPG group, which demonstrated dense, well-organized trabecular bone and greater defect filling. Similarly, the OPG group showed improved bone formation compared to single-treatment groups, although to a lesser extent than TPG.

**Figure 4:**
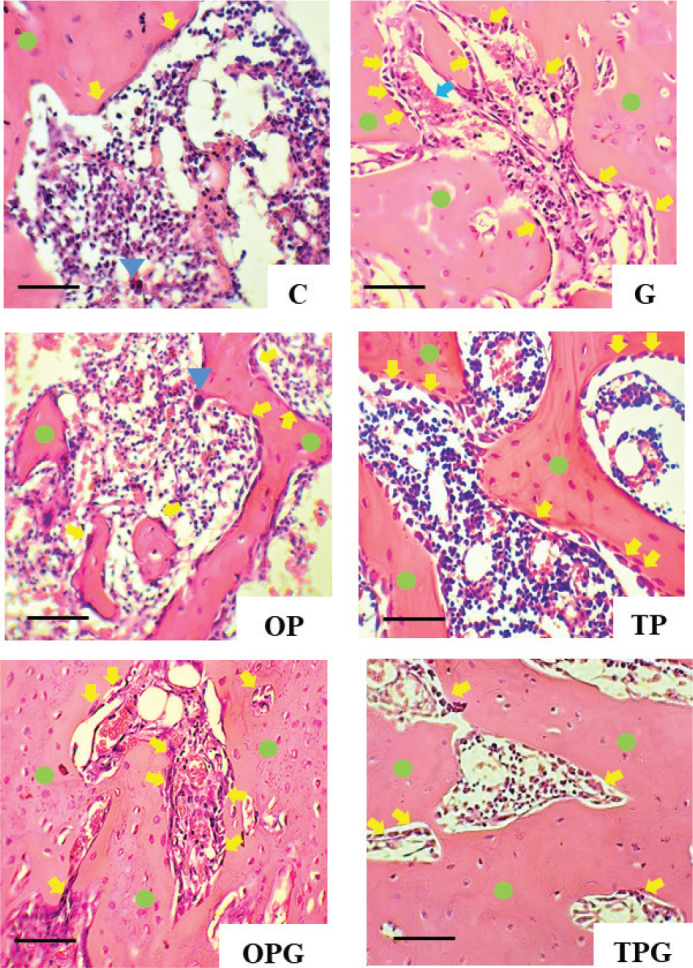
Histological samples appearances at 100× magnification. Yellow arrows indicate osteoblasts, blue triangles indicate osteoclasts, blue arrows indicate residual xenograft materials, and green circles indicate new bone sequestra. (Scale bar: 50 µm).

Histological evaluation at 100× magnification (Figure 4) showed clear differences in cellular activity and bone remodeling among groups. The C group had limited osteogenic activity, with few osteoblasts, scattered osteoclasts, and minimal new bone formation; the defect area remained mostly filled with fibrous and hematopoietic tissue. The G group showed active bone remodeling, with osteoblasts lining newly formed trabeculae, scattered osteoclasts, and residual xenograft material within the developing bone matrix. The OP group showed more osteoblasts along trabecular surfaces and moderate new bone formation, indicating greater osteoblastic activity than the control. The TP group exhibited pronounced bone remodeling, with higher osteoblast density, more organized trabeculae, and active osteoclasts, suggesting ongoing remodeling.

The combination groups showed the most advanced regeneration. The OPG group had substantial new bone formation, increased osteoblast activity, and residual xenograft particles integrated within the new bone matrix. The TPG group displayed the most mature bone architecture, with dense, organized trabeculae, abundant osteoblasts, and reduced osteoclast activity, indicating advanced bone remodeling. Minimal residual xenograft material and prominent new bone sequestra suggested effective graft integration and accelerated osteogenesis.

### Histomorphometric evaluation

**Figure 5:**
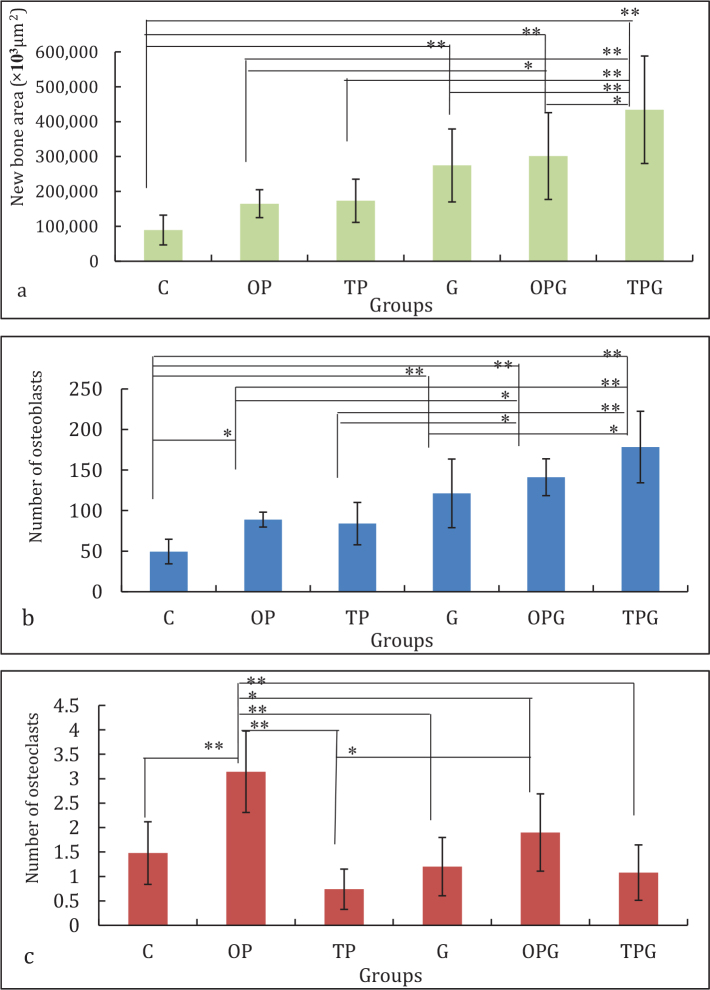
(a) New bone area, (b) number of osteoblasts, and (c) number of osteoclasts of the experimental group in the tibia defect after 4 weeks of healing period. Statistical difference *p* < 0.01 (**) and *p* < 0.05 (*).

Histomorphometric analysis at 4 weeks (Figure 5) showed significant differences in new bone formation and cellular activity among groups. New bone area increased progressively from the control group to the combination treatments, with mean values (µm²) of 89,408 ± 42,376 (C), 164,903 ± 39,741 (OP), 173,394 ± 61,974 (TP), 274,553 ± 104,339 (G), 301,517 ± 124,401 (OPG), and 434,196 ± 153,911 (TPG). The TPG group had the highest new bone area, significantly exceeding C, OP, TP, and G (*p* < 0.01). The OPG group also differed significantly from OP (*p* < 0.05) and control (*p* < 0.01).

Osteoblast counts followed a similar trend, increasing from 49.4 ± 15.2 (C) to 178.4 ± 44.2 (TPG). The TPG group exhibited significantly higher osteoblast numbers than all other groups (*p* < 0.01), while OPG was significantly higher than OP and TP (*p* < 0.05). Compared with control, all treatment groups showed increased osteoblast activity, with statistically significant differences observed for G, OPG, and TPG (*p* < 0.01).

Osteoclast counts were generally lower and more regulated in the combination groups. The OP group showed the highest osteoclast count (3.1 ± 0.8), significantly greater than all other groups (*p* < 0.01). In contrast, TP (0.7 ± 0.4) and TPG (1.1 ± 0.6) exhibited lower osteoclast levels, indicating reduced resorptive activity. Significant differences were observed between OP and OPG, TP, and TPG (*p* < 0.01), as well as between TP and OPG (*p* < 0.05).

The C group had the lowest new bone area and osteoblast count, indicating limited regeneration and minimal remodeling. The OP group showed moderate increases in new bone area and osteoblasts, with higher osteoclast counts, suggesting early-stage remodeling. The TP group demonstrated further improvement, with more new bone and osteoblasts than OP, and lower osteoclast activity, indicating a shift toward bone formation. The G group had moderate increases in new bone and osteoblasts compared to control, with balanced osteoclast activity, reflecting active remodeling and partial graft integration. The combination groups showed the strongest regenerative effects. The OPG group had substantial increases in new bone and osteoblasts compared to single treatments, with moderate osteoclast activity, indicating enhanced turnover and graft integration. The TPG group had the highest new bone area and osteoblast numbers, with controlled osteoclast levels, indicating advanced bone formation and maturation.

### Blood glucose and hemoglobin evaluation

**Figure 6:**
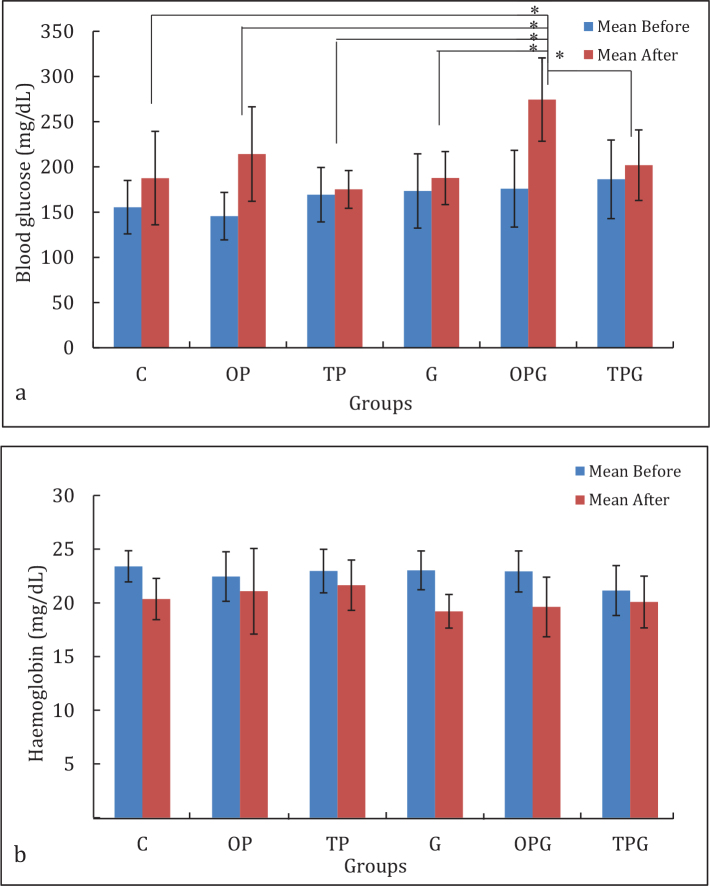
(a) Blood glucose and (b) hemoglobin measurement before and after a 4-week healing period. Statistical difference *p* < 0.01 (**) and *p* < 0.05 (*).

Systemic parameter analysis revealed changes in blood glucose and hemoglobin levels before and after the 4-week healing period in all groups (Figure 6). The C group showed a slight increase in blood glucose and a mild decrease in hemoglobin after healing. The OP group had a greater increase in blood glucose and a moderate reduction in hemoglobin. The TP group showed a modest rise in blood glucose, while hemoglobin levels remained stable. The G group showed a moderate increase in blood glucose and a noticeable decline in hemoglobin. The OPG group had the greatest increase in blood glucose and the greatest reduction in hemoglobin. The TPG group demonstrated a moderate increase in blood glucose, while hemoglobin levels remained relatively stable.

## Discussion

This study evaluated the effects of oral and topical propolis administration, alone and in combination with a xenograft biomaterial, on bone healing in a standardized tibial defect model. All treatment groups showed a significant enhancement in bone regeneration compared with the control, as demonstrated by radiographic, histological, and histomorphometric analyses. Notably, combination therapies, particularly topical propolis with xenograft (TPG), produced the most favorable outcomes. The TPG group achieved the highest bone density (81.09%), the largest new bone area (434,195.7 µm²), increased osteoblast numbers, and regulated osteoclast activity. These results demonstrate that combining bioactive natural compounds with osteoconductive scaffolds enhances bone regeneration [25], a key aspect of bone tissue engineering [26]. Previous studies reported only moderate increases in bone density with biomaterials alone (35–45% in xenograft-only groups after 8 weeks) [27], whereas the TPG group in this study showed significantly greater improvement, underscoring the benefits of adding propolis. Similarly, Shete et al. reported increased bone density with natural compound-biomaterial combinations, highlighting the stronger effect of topical propolis [28]. Furthermore, while Uçan et al. found that systemic propolis increased density by approximately 30% in rat defects, the topical approach in this study yielded superior results, demonstrating the advantage of local delivery [29].

Radiographic analysis indicated increased bone density in all treatment groups, with the TPG group exhibiting a more than 4-fold increase compared to the control (19.11%). These findings suggest enhanced mineral deposition and accelerated bone maturation. The observed hierarchy of effectiveness (C < OP < TP < G < OPG < TPG) demonstrates that both the administration route of propolis and its combination with xenograft significantly affect regenerative outcomes. These results align with the established roles of osteogenesis, osteoinduction, and osteoconduction, in which xenografts serve as structural scaffolds, and propolis provides bioactive signals that promote bone formation [30]. Although the ImageJ-based panoramic radiograph method provides only RBD values and has limitations related to two-dimensional imaging, machine settings, and the object positioning, it demonstrated high reproducibility and standardized assessment [24]. Therefore, it serves as a practical, accessible, and scientifically reliable alternative to micro-CT for comparative cross-sectional bone density evaluation in clinical research.

Histological analysis revealed well-organized and interconnected trabecular structures in the TPG group, in contrast to the disorganized patterns typically observed during early remodeling with scaffold-only approaches [31]. Additionally, the inflammatory response was significantly reduced in the combination groups, indicating a protective and modulatory effect. This observation is consistent with the established anti-inflammatory properties of propolis [29] and its components, particularly caffeic acid phenethyl ester (CAPE), Nuclear Factor kappa-light-chain-enhancer of activated B cells (NF-kB) signaling [29] and reduces pro-inflammatory cytokines [32], thereby suppressing osteoclastogenesis and reducing bone resorption [33].

At the cellular level, enhanced regenerative outcomes are attributable to increased osteoblast activity and regulated osteoclast function. The combination groups demonstrated higher osteoblast counts, reflecting stimulation of bone-forming cells [33], while osteoclast activity remained controlled. Achieving a balance between bone formation and resorption is critical for optimal healing. The underlying biological mechanisms are associated with propolis’s complex composition, as flavonoids activate signaling pathways that are essential for osteoblast differentiation and proliferation [34].

The anti-inflammatory and antioxidant properties of propolis further contribute to its regenerative potential. Chronic inflammation and oxidative stress impair bone healing by promoting osteoclast activity and inhibiting osteoblast function [35]. Propolis reduces pro-inflammatory cytokines and enhances antioxidant defenses. In this study, reduced inflammatory cell infiltration was observed in the treated groups, especially in the combination group, indicating a more favorable microenvironment for bone regeneration [32]. Previous study reported that propolis extract contains phenolics such as chlorogenic acid and 4-methoxycinnamic acid, flavonoids including apigetrin, apigenin, luteolin, diosmetin, baicalin, rhoifolin, and scutellarin, and coumarins such as 7-hydroxycoumarine. It also includes anti-inflammatory compounds, particularly α,β-amyrin and lupeol, which highlight its potential for health and medicinal applications [36].

Xenografts serve as essential osteoconductive scaffolds in this context. Their porous structure supports cell attachment, proliferation, and vascularization, but they possess limited osteoinductive capacity when used alone [26]. The addition of propolis introduces bioactive signals that enhance osteogenesis and improve regenerative outcomes [35]. The comparison between oral and topical administration underscores the significance of delivery strategy. While both methods improved bone healing compared to the control, topical application consistently resulted in higher bone density, increased new bone formation, and greater osteoblast activity.

Systemic evaluation showed that blood glucose levels increased after the 4-week healing period in all experimental groups, with the highest rise in the OPG group. However, glucose values remained within the physiological range for healthy rats (approximately 100–300 mg/dL), indicating that neither propolis nor xenograft caused pathological hyperglycemia [37]. This aligns with previous studies suggesting that propolis modulates glucose metabolism through antioxidant and anti-inflammatory effects, thereby improving insulin sensitivity and supporting glucose homeostasis [38, 39]. Hemoglobin levels decreased slightly after treatment in most groups but stayed within the normal range for rats (approximately 12–18 g/dL), indicating no adverse hematological effects [40]. The stable hemoglobin levels in the combination groups, particularly TPG, further support the systemic safety of propolis–xenograft therapy. These results are consistent with earlier reports that propolis does not negatively affect hematological parameters and may protect against oxidative stress-induced erythrocyte damage [41, 42].

These findings have important clinical implications for regenerative dentistry and implantology. Bone resorption after tooth extraction remains a challenge that can limit implant placement. While xenografts are widely used for ridge preservation, their regenerative capacity may be enhanced by adding bioactive compounds [43]. The results suggest that combining propolis with xenograft, especially through topical application, can improve both the quantity and quality of regenerated bone. However, several limitations should be considered. Animal models do not fully represent human bone biology, and metabolic differences may affect clinical translation [26]. The short observation period only captures early-stage healing and does not address long-term bone stability. Additionally, the composition of propolis varies by geographical and botanical origin [43], highlighting the need for standardization in future applications. It is important to recognize that tibial cortical bone and alveolar bone are fundamentally distinct structures, differing considerably in anatomy, vascular supply, mechanical loading environment, and healing mechanisms. Therefore, the findings of this study cannot be directly applied to clinical alveolar conditions, such as post-extraction socket resorption or pre-implant bone augmentation, and any extrapolation to such contexts should be approached with considerable caution. Future research should prioritize optimizing propolis delivery systems, including incorporation into scaffolds, nanoparticles, or hydrogels, to achieve controlled and sustained release. Combining propolis with growth factors, such as bone morphogenetic proteins (BMPs), or with stem cell-based therapies may further enhance regenerative outcomes. Long-term and clinical investigations are required to validate these findings and establish standardized protocols for clinical application.

## Conclusion

Combining propolis extract with xenograft enhances bone regeneration in rat tibial defects, as shown by increased osteoblast activity and new bone formation compared to either treatment alone or the control group. The xenograft acts as a scaffold for bone growth, while propolis supplies compounds that stimulate bone formation. Topical application of propolis was more effective than oral administration, likely because it delivers active ingredients directly to the injury site. These findings indicate that propolis may be a valuable addition to bone regeneration protocols before dental implant placement. However, further research is required to establish optimal dosage, delivery methods, and clinical applications of propolis-based therapies in regenerative dentistry.

## Data Availability

The datasets generated and/or analyzed during the current study are not publicly available but may be obtained from the corresponding author upon reasonable request.
